# Broadband Achromatic Metasurfaces for Longwave Infrared Applications

**DOI:** 10.3390/nano11102760

**Published:** 2021-10-18

**Authors:** Naitao Song, Nianxi Xu, Dongzhi Shan, Yuanhang Zhao, Jinsong Gao, Yang Tang, Qiao Sun, Xin Chen, Yansong Wang, Xiaoguo Feng

**Affiliations:** 1 Key Laboratory of Optical System Advanced Manufacturing Technology, Changchun Institute of Optics, Fine Mechanics and Physics, Chinese Academy of Sciences, Changchun 130033, China; songnaitao@ciomp.ac.cn (N.S.); xnxlzhy999@126.com (N.X.); jlxjusdz@163.com (D.S.); tangyang@163.com (Y.T.); sqsuqiao@126.com (Q.S.); chexin_19344834@163.com (X.C.); wangyansong99@163.com (Y.W.); fxg74@163.com (X.F.); 2 College of Da Heng, University of the Chinese Academy of Sciences, Beijing 100039, China; zhaoyhciomp@163.com; 3 Jilin Provincial Key Laboratory of Advanced Optoelectronic Equipment and Instrument Manufacturing Technology, Changchun 130033, China

**Keywords:** achromatic metalens, achromatic metasurface grating, longwave infrared, dynamic phase, Pancharatnam–Berry phase

## Abstract

Longwave infrared (LWIR) optics are essential for several technologies, such as thermal imaging and wireless communication, but their development is hindered by their bulk and high fabrication costs. Metasurfaces have recently emerged as powerful platforms for LWIR integrated optics; however, conventional metasurfaces are highly chromatic, which adversely affects their performance in broadband applications. In this work, the chromatic dispersion properties of metasurfaces are analyzed via ray tracing, and a general method for correcting chromatic aberrations of metasurfaces is presented. By combining the dynamic and geometric phases, the desired group delay and phase profiles are imparted to the metasurfaces simultaneously, resulting in good achromatic performance. Two broadband achromatic metasurfaces based on all-germanium platforms are demonstrated in the LWIR: a broadband achromatic metalens with a numerical aperture of 0.32, an average intensity efficiency of 31%, and a Strehl ratio above 0.8 from 9.6 μm to 11.6 μm, and a broadband achromatic metasurface grating with a constant deflection angle of 30° from 9.6 μm to 11.6 μm. Compared with state-of-the-art chromatic-aberration-restricted LWIR metasurfaces, this work represents a substantial advance and brings the field a step closer to practical applications.

## 1. Introduction

The longwave infrared (LWIR) wavelength band from 8 μm to 12 μm is essential for a wide range of applications, including environmental sensing, medical imaging, wireless communication, and nighttime autonomous driving [1,2,3]. For example, at international airports, LWIR thermal imaging is often used to identify fevers in travelers, to contain the spread of deadly infectious diseases. However, conventional LWIR optics are bulky and expensive compared with their visible and near-infrared counterparts, which hinders the further development of LWIR integrated optics.

Metasurfaces comprising subwavelength meta-atoms can locally control the phase, polarization, and amplitude of light, and are promising platforms for integrated optical devices. In 2011, Yu et al. [4] first demonstrated a metasurface that deflects light anomalously through an aperiodic array of V-shaped plasmonic antennas. Since then, plasmonic metasurfaces have been widely investigated, and various plasmonic metadevices based on the resonance phase have been demonstrated [5,6,7]. However, the efficiency of plasmonic metasurfaces is greatly limited owing to the absorption loss of metallic material. To solve this dilemma, dielectric metasurfaces composed of high-refractive-index dielectric antennas are used to realize optical metadevices with high efficiency [8,9]. Moreover, the dielectric metasurface can be processed at low cost using standard nanofabrication approaches. To date, various metasurfaces with extraordinary functionalities have been demonstrated, such as metalenses [4,10,11,12,13], metasurface gratings [14,15], orbital angular momentum (OAM) generators [16,17], retroreflectors [18,19,20], metaholograms [21,22,23], structured light generators [24,25], and nonlinear optics [26], covering a wide spectrum from ultraviolet to microwave wavelengths. Recently, metasurfaces have been employed in biosensing applications [27]. By integrating a metasurface on the tip of an optical fiber, highly sensitive detection of biological substances has been achieved [28]. Conventional metadevices are highly chromatic, despite comprising weakly dispersive materials. Some pioneering studies have demonstrated achromatic metasurfaces at discrete multiwavelengths or narrow wavebands [29,30]. However, broadband achromatic metasurfaces are more desirable owing to their compact size and powerful functionality. Chen et al. [31] reported a broadband achromatic TiO_2_ metalens in the visible region; Wang et al. [32] reported a broadband achromatic Au metalens and Au metasurface grating in the near-infrared range; Ou et al. [33] reported broadband achromatic focusing vortex generators based on all-silicon platforms in the mid-infrared regime. 

Studies on LWIR metasurfaces are rather scarce compared with metasurfaces at other wavelengths [34,35,36]. A significant reason for this is the lack of available materials, since most optical materials (e.g., silicon and silicate glasses) are opaque in the LWIR regime. In addition, most achromatic metasurfaces take the form of high-refractive-index material patterns at wavelength-scale heights on low-refractive-index substrates [13,37,38,39,40]. However, depositing wavelength-thickness high-refractive-index films in the LWIR range is challenging owing to quality issues and material stress. Van der Waals materials, such as MoO_3_, offer new opportunities for infrared metaphotonics [41,42,43,44,45]. In this study, we propose a general method of implementing LWIR achromatic metasurfaces based on all-germanium platforms. To demonstrate the validity of the proposed method, a broadband achromatic metalens (BAML) and a broadband achromatic metasurface grating (BAMG) operating in the LWIR range are presented.

The remainder of this work is organized as follows. First, we analyze the dispersion properties of chromatic metasurfaces via ray tracing. Then, we summarize the general conditions for correcting chromatic aberrations and introduce a method to satisfy these conditions by manipulating the phase and group delays of birefringent meta-atoms simultaneously. Finally, we present a BAML with a diameter of 400 μm, a numerical aperture (NA) of 0.32, and an average intensity efficiency as high as 31%, operating from 9.6 μm to 11.6 μm. Further analysis of the Strehl ratio confirms the achromatic diffraction-limited focusing performance of the proposed BAML. To demonstrate the versatility of the proposed method, a BAMG with a constant deflection angle of 30° from 9.6 μm to 11.6 μm is also implemented. To the best of our knowledge, this study presents the first reported transmission broadband achromatic metasurfaces for LWIR. Moreover, owing to the low loss and high refractive index of germanium, the efficiency of the proposed broadband achromatic metasurface is as high as those of the monochromatic metasurfaces reported in the literature [35,46]. We believe that the proposed work represents a substantial advance and brings the field a step closer to practical applications.

## 2. Principles and Design

### 2.1. Chromatic Dispersion of a Metasurface

#### 2.1.1. Chromatic Dispersion of a Metasurface Grating

**Figure 1:**
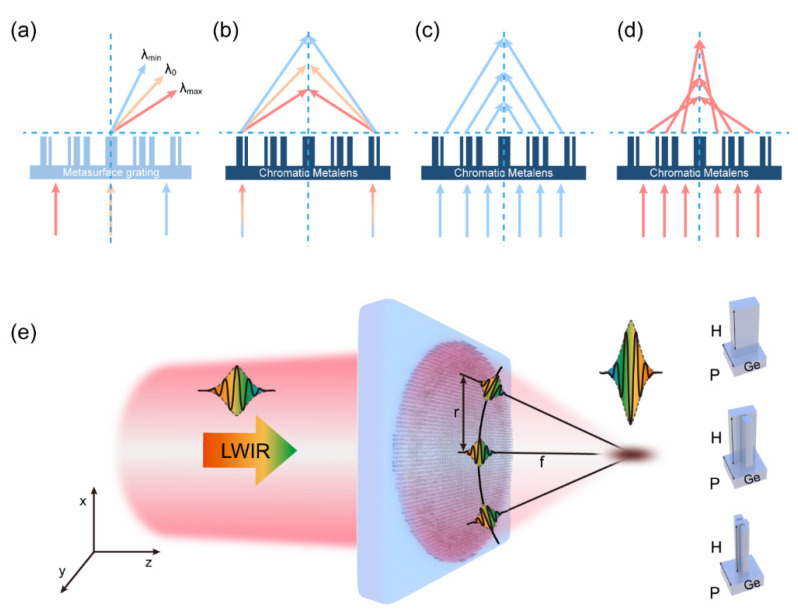
Dispersion properties of chromatic metasurfaces and schematic of the achromatic metalens. (**a**) Angular dispersion of the metasurface grating; (**b**) dispersion of polychromatic light incident at the edge of the chromatic metalens; (**c**,**d**) ray tracing of light at different coordinates; (**e**) schematic of the achromatic metalens in the LWIR range. In (**a**) through (**d**), the red ray represents light at $\lambda_{\max}$, the yellow ray represents light at $\lambda_{0}$, and the blue ray represents light at $\lambda_{\min}$. On the right is a schematic of meta-atoms, where H is the height of the nanopillar, and *p* is the period of the nanopillar. All meta-atoms have the same height and period.

A metasurface is characterized by a local phase gradient that results in a local deflection angle. A metasurface grating is a type of device whose local phase gradient is constant across its surface. The behavior of a metasurface is governed by the generalized Snell’s principle, which can be derived from Fermat’s principle [4]. For a metasurface grating under normal illumination, the generalized Snell’s principle is of the form

(1) $$\frac{2\pi }{\lambda }\sin\left(\theta \right)=k_{G}$$

where *θ* is the deflection angle, *λ* is the wavelength, and $k_{G}=\frac{\partial \varphi }{\partial x}$ is the local grating momentum or phase gradient. For simplicity, we assume that the phase gradient is introduced by the Pancharatnam–Berry (PB) phase modulation only [47]; therefore, the phase gradient is independent of the wavelength, and the angular dispersion relation of the grating can be obtained simply, as follows:

(2) $$\theta =\arcsin\left(\frac{\lambda }{\lambda_{0}}\sin\left(\theta_{0}\right)\right)$$

where $\theta_{0}$ is the deflection angle at the central wavelength $\lambda_{0}$. As depicted in Figure 1a, larger deflection angles are observed at longer wavelengths, and an identical phenomenon occurs when polychromatic light passes through a prism composed of a material with negative dispersion. Therefore, we also refer to the dispersion of the metasurface grating as “negative chromatic dispersion”.

#### 2.1.2. Dispersion of a Metalens

A metalens is a type of metasurface that can focus light on a diffraction-limited spot. The local deflection angle at each point on the metalens depends on the radial coordinate as follows:

(3) $$\frac{-r}{f}=\tan\theta$$

where *f* is the focal length, *r* is the radial coordinate, and *θ* is the local deflection angle. Considering the relationship between the deflection angle and the wavelength in Equation (2), the relationship between the focal length at coordinate *r* and the wavelength can be expressed as

(4) $$f=\frac{f_{0}}{\cos\left(\theta_{0}\right)}\sqrt{{\left(\frac{\lambda_{0}}{\lambda }\right)}^{2}-\sin^{2}\left(\theta_{0}\right)}$$

where $f_{0}$ and $\theta_{0}$ are the focal length and the local deflection angle at the central wavelength, respectively. As depicted in Figure 1b, the focal point is closer to the metalens at a longer wavelength. Moreover, using the geometric relationship $\cos\left(\theta_{0}\right)=\frac{f_{0}}{\sqrt{r^{2}+f_{0}^{2}}},\;\sin\left(\theta_{0}\right)=\frac{-r}{\sqrt{r^{2}+f_{0}^{2}}}$, Equation (4) can be rewritten as

(5) $$f=\sqrt{\left(\frac{\lambda_{0}^{2}-\lambda^{2}}{\lambda^{2}}\right)r^{2}+\frac{\lambda_{0}^{2}}{\lambda^{2}}f_{0}^{2}}$$

As depicted in Figure 1c,d, the focal length is radially dependent at wavelengths other than the central wavelength, indicating that the metalens is no longer free of spherical aberrations. Specifically, when $\lambda <\lambda_{0}$ a positive spherical aberration is introduced, and when $\lambda >\lambda_{0}$ a negative spherical aberration is introduced. Notably, this phenomenon is more severe in high-NA metalenses than in low-NA metalenses (where $r\ll f_{0}$). In addition, for low-NA metalenses, Equation (5) can be approximately simplified as $f\lambda =f_{0}\lambda_{0}$, indicating that the focal length is inversely proportional to the wavelength, which helps to estimate the longitudinal chromatic aberrations of the metalenses. Briefly, chromatic dispersion of a metalens may cause defocusing and spherical aberrations, both of which adversely affect its performance.

Here, it is again emphasized that the dispersion law of the chromatic metasurface obtained above is for the case where an abrupt phase is introduced only by PB phase modulation; the dispersion law for the case where an abrupt phase is introduced by resonance is more complex and cannot be predicted directly from the ray-tracing perspective.

### 2.2. Achromatic Metasurface Design

Without loss of generality, we derive the principle of the BAML. According to the discussion in Section 2.1, to realize broadband achromatic diffractive limit focusing, the local deflection angle should not vary with wavelength. Therefore, the phase gradient imparted to the metalens must satisfy the following equation:

(6) $$\frac{\partial \varphi \left(r,\omega \right)}{\partial r}=\frac{2\pi }{\lambda }\frac{-r}{\sqrt{r^{2}+f_{0}^{2}}}=\frac{\omega }{c_{0}}\frac{-r}{\sqrt{r^{2}+f_{0}^{2}}}$$

where *ω* is the angular frequency, and $c_{0}$ is the speed of light in a vacuum. Equation (6) shows that the phase gradient is linearly proportional to the frequency when the deflection angle does not vary with frequency. We can integrate Equation (6) from the edge of the metalens (*r* = *R*_max_) to an arbitrary point (*r* = *R*) on the metalens, such that the phase at *r* = *R* is of the form

(7) $$\varphi \left(R,\omega \right)=\frac{\omega }{c_{0}}\left(\sqrt{R_{\max}^{2}+f_{0}^{2}}-\sqrt{R^{2}+f_{0}^{2}}\right)+\varphi \left(R_{\max},\omega \right)$$

This phase profile is hyperbolic at any given frequency, and the phase dispersion is determined by the phase dispersion of the reference point (*r* = *R*_max_). In the general case, $\varphi \left(R_{\max},\omega \right)$ can be Taylor expanded around the central frequency, as

(8) $$\begin{array}{r} \varphi \left(R_{\max},\omega \right)=\varphi \left(R_{\max},\omega_{0}\right)+\left(\omega -\omega_{0}\right){\frac{\partial \varphi \left(R_{\max},\omega \right)}{\partial \omega }|}_{\omega =\omega_{0}}\\ +\frac{1}{2}{\left(\omega -\omega_{0}\right)}^{2}{\frac{\partial^{2}\varphi \left(R_{\max},\omega \right)}{\partial \omega^{2}}|}_{\omega =\omega_{0}}+\ldots \end{array}$$

where $\omega_{0}\;$ is the central angular frequency, and the first three terms on the right-hand side (RHS) of Equation (8) are the phase, group delay, and group delay dispersion, respectively. Theoretically, the phase dispersion at the reference point can be arbitrary; however, when the third term and remaining terms on the RHS of Equation (8) are not zero, the operating bandwidth of the BAML will be limited. Thus, to achieve broadband achromatic performance, we retain only the first two terms on the RHS of Equation (8), meaning that the phase of the reference point is linearly proportional to the frequency. By substituting this result in Equation (7), we find that the phase at any point on the BAML is linear with respect to the frequency, and that the slope of the phase with respect to the frequency and the phase at the central frequency point are determined by the following equations, respectively:

(9) $$\frac{\partial \varphi \left(R,\omega \right)}{\partial \omega }={\frac{1}{c_{0}}\left(\sqrt{R_{\max}^{2}+f_{0}^{2}}-\sqrt{R^{2}+f_{0}^{2}}\right)+\frac{\partial \varphi \left(R_{\max},\omega \right)}{\partial \omega }|}_{\omega =\omega_{0}}$$

(10) $$\varphi \left(R,\omega_{0}\right)=\frac{\omega_{0}}{c_{0}}\left(\sqrt{R_{\max}^{2}+f_{0}^{2}}-\sqrt{R^{2}+f_{0}^{2}}\right)+\varphi \left(R_{\max},\omega_{0}\right)$$

At this point, we have summarized the two conditions for correcting the chromatic aberrations of the BAML: the group delay (derivative of the phase with respect to frequency) condition as shown in Equation (9), and the phase condition as shown in Equation (10). The physical insights into these conditions are illustrated in Figure 1e, where a light pulse is assumed to be incident from the left side of the metalens, and the group delay condition of Equation (8) ensures that the group delay acquired by the metalens compensates for the time delay caused by the differences in optical path length (group delay is equal to the actual time delay experienced by the signal in most cases [48]). Therefore, plane-wave packets incident from any radial coordinate on the metalens reach the focal point at the same time. The phase condition of Equation (9) ensures that the plane-wave packets incident from any radial coordinate arrive at the focal point with the same phase; thus, they can interfere constructively.

Anisotropic nanostructures are generally utilized to modulate both the phase and group delays, as shown in Figure 1e. Owing to the anisotropic geometry, such nanostructures can be treated as birefringent wave plates. When circularly polarized light is incident on such anisotropic nanostructures, both dynamic and PB phases are introduced in the cross-polarized component of the electric field (light). The dynamic phase is frequency dependent, whereas the PB phase is frequency independent; hence, the phase and group delays can be tuned independently.

The dependence of the outgoing light on the incoming light can be described using the Jones calculus as follows:

(11) $$\left[\begin{array}{c} E_{x}^{out}\\ E_{y}^{out} \end{array}\right]=R\left(\theta \right)\cdot M\cdot R\left(-\theta \right)\left[\begin{array}{c} E_{x}^{in}\\ E_{y}^{in} \end{array}\right]$$

where R(θ) is a rotation matrix that can be expressed as $R\left(\theta \right)=\left[\begin{array}{cc} \cos\theta & \sin\theta \\ -\sin\theta & \cos\theta \end{array}\right]$, θ is the rotation angle with respect to the *x* axis, and $M=\left[\begin{array}{cc} A_{o}e^{i\varphi_{o}} & 0\\ 0 & A_{e}e^{i\varphi_{e}} \end{array}\right]$ is a matrix that accounts for the amplitudes ($A_{o}$ and $A_{e}$) and phases ($\varphi_{o}$ and $\varphi_{e}$) of ordinary light (polarized along the *x* axis) and extraordinary light (polarized along the *y* axis), respectively. The relation between the outgoing light and incoming light can be rewritten as

(12) $$\left[\begin{array}{c} E_{x}^{out}\\ E_{y}^{out} \end{array}\right]=\left(\frac{A_{o}e^{i\varphi_{o}}+A_{e}e^{i\varphi_{e}}}{2}\left[\begin{array}{cc} 1 & 0\\ 0 & 1 \end{array}\right]+\frac{A_{o}e^{i\varphi_{o}}-A_{e}e^{i\varphi_{e}}}{2}\left[\begin{array}{cc} \cos2\theta & \sin2\theta \\ \sin2\theta & -\cos2\theta \end{array}\right]\right)\left[\begin{array}{c} E_{x}^{in}\\ E_{y}^{in} \end{array}\right]$$

Here, we assume that the incident light is left-circularly polarized (LCP) and injected from the bottom of the substrate:

(13) $$\left[\begin{array}{c} E_{x}^{out}\\ E_{y}^{out} \end{array}\right]=\frac{A_{o}e^{i\varphi_{o}}+A_{e}e^{i\varphi_{e}}}{2}\frac{1}{\sqrt{2}}\left[\begin{array}{c} 1\\ i \end{array}\right]+\frac{A_{o}e^{i\varphi_{o}}-A_{e}e^{i\varphi_{e}}}{2}e^{i2\theta }\frac{1}{\sqrt{2}}\left[\begin{array}{c} 1\\ -i \end{array}\right]$$

Equation (13) indicates that the phase of the outgoing right-circularly polarized (RCP) light can be divided into two parts. The first part is $\;\varphi_{d}=\arg\left(A_{o}e^{i\varphi_{o}}-A_{e}e^{i\varphi_{e}}\right)$, known as the dynamic phase, where arg denotes the argument of the complex number, $\varphi_{o}=\frac{\omega }{c_{0}}n_{o}h$, and $\varphi_{e}=\frac{\omega }{c_{0}}n_{e}h,$ where h is the height of the nanostructure, and $n_{o},n_{e}$ are the effective refractive indices experienced by the ordinary and extraordinary light, respectively. The second part is $\varphi_{g}=2\theta$, known as the PB phase. It is seen that the dynamic phase is frequency dependent and the PB phase is frequency independent; thus, by combining the dynamic and PB phases, we can manipulate both the group and phase delays. Moreover, when $\varphi_{o}-\varphi_{e}=\pi$, the amplitude of the outgoing RCP light $A_{rcp}=\mathrm{abs}\left(\frac{A_{o}e^{i\varphi_{o}}-A_{e}e^{i\varphi_{e}}}{2}\right)$ is maximized, and the nanostructure behaves as a half-wave plate. We define the cross-polarization conversion ratio (PCR) as $\eta =\frac{4A_{rcp}^{2}}{A_{o}^{2}+A_{e}^{2}}\times 100\%$ to characterize the intensity proportion of cross polarization in the outgoing light. Because the PB phase only acts on the cross-polarization component, the PCR must be as large as possible so that more of the outgoing light is converted to the cross-polarization component.

According to the discussion above, the design of the achromatic metalens can be generally divided into four steps. First, the target phase and group delays are calculated according to Equations (9) and (10). Second, a library of nanostructures should be built to connect a given structural geometry to the dynamic phase that it provides. Third, a linear fitting is conducted on the dynamic phase spectrum of each nanostructure in the library, and the group delay is calculated. Finally, the target group delay profile is matched by identifying the nanostructure that imparts a group delay that is as close to the target one as possible, and the target phase profile is matched by rotating the selected nanostructure to impart a frequency-independent PB phase.

## 3. Results and Discussion

### 3.1. Meta-Atom

Although the geometric parameters play an important role in the design of nanostructures, their properties are also fundamentally determined by the constituent materials. Owing to well-developed fabrication techniques and complementary metal-oxide-semiconductor (CMOS) compatibility, silicon is usually chosen as the base material for infrared (IR) metasurfaces [35,36,49,50]; however, silicon shows strong absorption beyond 8.5 μm [51]. In this study, we chose monocrystalline germanium as the base material. On the one hand, germanium has a high refractive index and low absorption loss in the LWIR range, which enables meta-atoms based on germanium to have strong light confinement, resulting in negligible interactions among the adjacent meta-atoms. On the other hand, the fabrication technique for germanium is also well developed; for example, various germanium-based platform (germanium-on-silicon and germanium-on-silicon-on-insulator) IR waveguides have been demonstrated [51].

**Figure 2:**
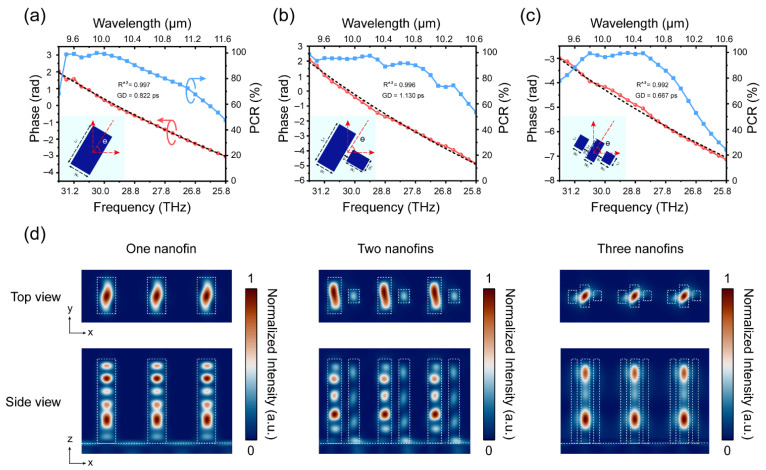
Simulation results for three selected meta-atoms. (**a**) PCR and phase spectrum for one nanofin, geometry parameters in the inset picture: L1 = 4.4 μm, W1 = 2.2 μm; (**b**) PCR and phase spectrum for two nanofins, geometry parameters in the inset picture: L1 = 4.3 μm, W1 = 1.3 μm, g1 = 0.5 μm, L2 = 1.9 μm, W2 = 1.9 μm; (**c**) PCR and phase spectrum for three nanofins, geometry parameters in the inset picture: L1 = 1.3 μm, W1 = 1 μm, g1 = 0.5 μm, L2 = 2.1 μm, W2 = 1 μm, L3 = 1.1 μm, W3 = 1 μm; (**d**) top and side views of normalized H field intensities for the three meta-atoms.

When light travels through a nanostructure, a portion of the light is confined inside the nanostructure, while the rest leaks into the surrounding material. This is similar to the situation occurring in a waveguide. Owing to this similarity, the optical properties of nanostructures can be better understood by treating them as miniature truncated waveguides. As illustrated in Figure 2, three meta-atom archetypes were employed in our library: one nanofin corresponds to a rectangular miniature waveguide, two nanofins correspond to slotted miniature waveguides, and three nanofins correspond to multi-slotted miniature waveguides. Extensive waveguide modes were supported by the meta-atoms in our library, which allowed more precise regulation of the group delays. Considering future processing constraints, we controlled the finest size of the nanostructures in the library to be no less than 1 μm. All the nanostructures had identical heights of *H* = 10 μm, which allowed an aspect ratio ≤10:1. The lattice constant *p* was equal to the spacing between the centers of the adjacent meta-atoms, and its inverse was equal to the sampling rate of the target phase. Therefore, for accurate and efficient implementation of the target phase profile, the Nyquist sampling criterion should be fulfilled; that is, the sampling rate should be larger than twice the highest spatial frequency of the phase ($\frac{1}{P}\geq 2NA/\lambda_{min}$ for BAML and $\frac{1}{P}\geq 2\sin\theta /\lambda_{min}$ for BAMG). The lattice constant should also be smaller than the shortest wavelength across the waveband, to suppress higher-order diffractions. Considering all the above, we set the lattice constant as *p* = 6.2 μm for all nanostructures. To obtain the phase spectrum, simulations were conducted using Lumerical’s FDTD solver (ANSYS Inc., Canonsburg, PA, USA) [52]. Periodic conditions were applied in the transverse direction, the perfect matched layer (PML) condition was applied in the longitudinal direction with respect to the propagation of light, and LCP illumination was from the substrate. We then performed a linear fitting for the phase spectra of all the meta-atoms in the library using a homemade linear regression program and screened the meta-atoms for an R-squared value greater than 0.95 and a mean PCR efficiency greater than 10%.

Figure 2a,c show the PCR and phase spectra for the three selected meta-atoms. The phases of the three meta-atoms were linear with respect to the frequency within the operating bandwidth, and the group delays of the three meta-atoms were 0.82 ps, 1.13 ps, and 0.67 ps, respectively. Figure 2d shows the top and side views of the normalized magnetic energy density in a periodic array for the three selected nanostructures. Owing to the waveguide-like effect, the light was observed to mostly remain within the nanostructures. This indicates that the group delay design for the elements was accurate even when arranged in a square lattice as a metasurface array; thus, the light coupling effects between adjacent pairs of elements can be ignored.

### 3.2. Broadband Achromatic Metalens

**Figure 3:**
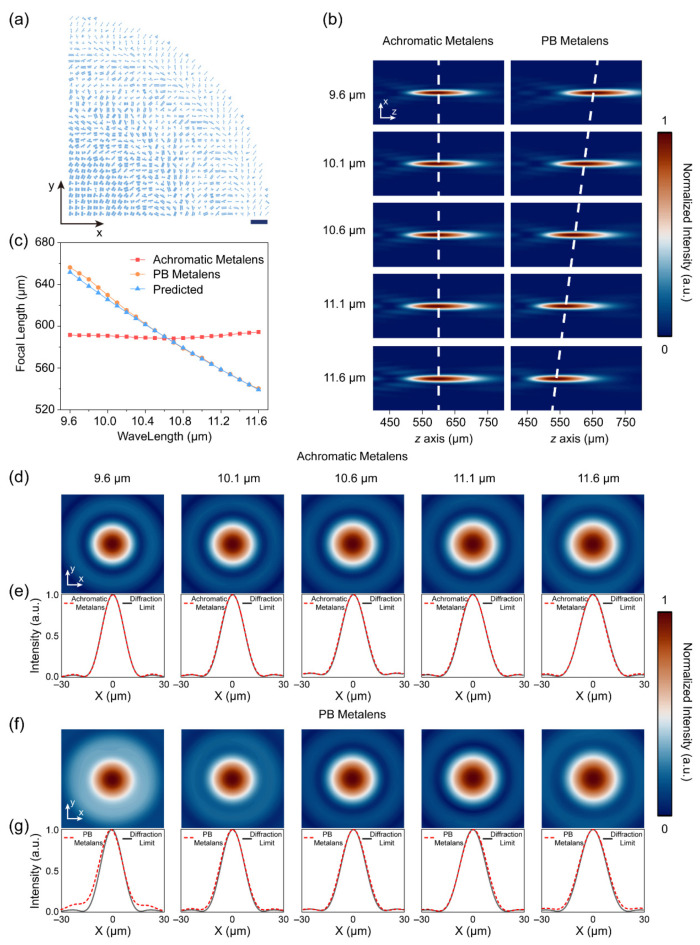
Layout and simulation results of intensity distributions of the metalenses: (**a**) layout of the broadband achromatic metalens, scale bar is 20 μm; (**b**) normalized intensity distribution in the x–z plane for the broadband achromatic metalens (left) and PB metalens (right) at a selected wavelength; (**c**) focal length as a function of the wavelength for the achromatic and PB metalenses, with the theoretically predicted focal length calculated as $f=\frac{\lambda_{0}f_{0}}{\lambda }$ for the PB metalens; (**d**,**f**) intensity distributions at the focal plane for the broadband achromatic metalens and PB metalens, respectively; (**e**,**g**) horizontal cuts (red dashed curves) across the focal spots in (**d**,**f**) compared with an ideal Airy spot (black curves).

Figure 3a shows the layout of our BAML with diameter *D* = 400 μm, NA = 0.32, and operating bandwidth from 9.6 μm to 11.6 μm. The PB metalens (whose layout is as shown in Figure S1) was composed of identical nanofins rotated according to the radial coordinates, and the PB metalenses were designed to have the same diameter and NA as the BAML at the central wavelength. Figure 3b presents the normalized intensity profile in the x–z plane and indicates that the focal point of the BAML did not shift with wavelength, whereas the focal point of the PB metalens moved toward the metalens as the wavelength increased. Figure 3c shows the extracted focal lengths of the BAML and PB metalens. The z coordinate corresponding to the peak intensity was considered to be the focal length for a given wavelength. The focal length of the BAML shifted from 0.32% to 0.65% relative to the mean focal length for the entire operating bandwidth, indicating the realization of a broadband achromatic converging property in the LWIR. As discussed in Section 2, the focal length of the PB metalens with low NA can be predicted by $f=\frac{\lambda_{0}f_{0}}{\lambda }$, and Figure 3c shows that the predicted and simulated focal lengths were in good agreement. 

We also characterized the metalenses in terms of their focal profiles. Figure 3d,f show the normalized intensity distributions at the focal plane (located at z = 590.5 μm) for the BAML and PB metalens, respectively. The PB metalens showed significant defocusing at 9.6 μm; in contrast, the focal spots of the BAML were diffraction limited for all wavelengths. Figure 3e,g show the corresponding normalized intensity distributions along the x-lines in Figure 3d,f, respectively. The intensity distribution of an ideal Airy disk is shown in Figure 3e,g for comparison. The intensity distribution of the achromatic metalens was very close to that of the ideal Airy disk, which confirms the excellent focusing performance of the BAML over the entire operating band.

**Figure 4:**
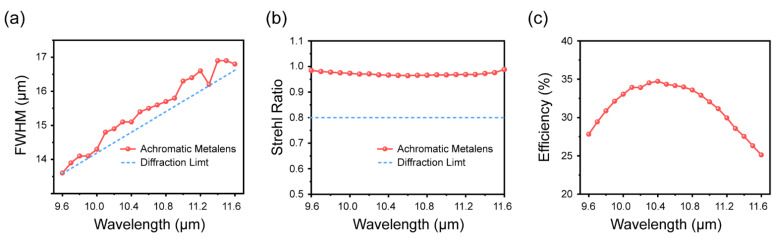
Simulation results of performance characterization of the broadband achromatic metalens. (**a**) FWHM for the broadband achromatic metalens, where the blue dashed line represents the theoretical FWHM of the ideal Airy disk; (**b**) Strehl ratio for the achromatic metalens, where the blue dashed line represents the diffraction-limited criterion; (**c**) intensity efficiency of the broadband achromatic metalens.

Figure 4a shows that the full width at half maximum (FWHM) of the BAML approached the diffraction limit ($0.257\times \frac{f\lambda }{D}$) at all wavelengths. The performance of the achromatic metalens was further quantified by calculating the Strehl ratio of the focal spots. The calculated Strehl ratios for the entire bandwidths of the metalenses are plotted in Figure 4b, and the definition of the Strehl ratio is the same as that in [30]. The Strehl ratios were above 0.8 for all wavelengths, thereby satisfying the condition for diffraction-limited focal spots. Figure 4c summarizes the intensity efficiency of the BAML. The intensity efficiency is defined as the ratio of optical power passing through a circular aperture (with a radius of two to three times the FWHM spanning the center of the focal spot) to the power incident on the metalens. The maximum intensity efficiency was 34%, and the average intensity efficiency was 31%. The efficiency of our BAML was close to that of the LWIR monochromatic metalens in [35]. The efficiency of the metalens can be further improved by increasing the PCR and scattering efficiency of the meta-atoms. The PCR of the meta-atoms can be improved by stacking several layers of nanofins, to work as an ideal achromatic wave plate [53], and the scattering efficiency of the meta-atoms can be improved by changing the substrate material to a low-refractive-index material, such as zinc selenide. 

### 3.3. Broadband Achromatic Metasurface Grating

**Figure 5:**
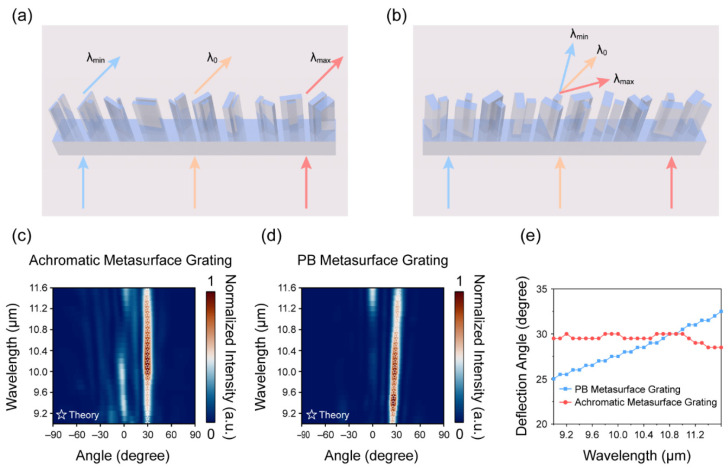
Schematic and deflection angles of the metasurface gratings: (**a**,**b**) schematic of the broadband achromatic and PB metasurface gratings, respectively; (**c**,**d**) far-field intensities of scattered light vs. angle of refraction for the broadband achromatic and PB metasurface gratings, respectively, where the stars in (**c**,**d**) represent the theoretically predicted deflection angles; (**e**) deflection angles of the broadband achromatic and PB metasurface gratings, where the deflection angle is defined as the angle corresponding to the maximum scattering intensities at the different wavelengths.

We designed and simulated a BAMG in the LWIR range to demonstrate the versatility of the proposed approach. Figure 5a shows a schematic of the proposed BAMG. The BAMG was composed of 10 elements with different in-plane geometry parameters, to satisfy the group delay condition:

(14) $${\frac{\partial \varphi \left(x,\omega \right)}{\partial \omega }|}_{\omega =\omega_{0}}=-\frac{x\sin(\theta_{0})}{c_{0}}+{\frac{\partial \varphi \left(0,\omega \right)}{\partial \omega }|}_{\omega =\omega_{0}}$$

Each element is rotated by a specific angle to introduce the PB phase to satisfy the phase condition:

(15) $$\varphi \left(x,\omega_{0}\right)=-\frac{x\sin(\theta_{0})\omega_{0}}{c_{0}}+\varphi \left(0,\omega_{0}\right)$$

For comparison, a PB metasurface grating (layout shown in Figure 5b) was composed of elements with identical geometry parameters but different rotation angles to match the phase condition. Figure 5c,d show the far-field intensities of the scattered light vs. the angle of refraction for the BAMG and PB metasurface grating, respectively. For the PB metasurface grating, high-intensity scattered light was concentrated at approximately 30° at most wavelengths. However, at 11.6 µm, the intensity of the scattered light near 0° was already comparable to that of the scattered light at 30°. This is mainly attributed to the reduced PCR (shown in Figure 2b) of the element comprising the PB grating. Considering the fact that the co-polarized component of the outgoing light is not deflected, whereas the cross-polarized component is anomalously deflected to the +1st-order diffraction direction, a higher PCR efficiency means that more scattered light is deflected to the 30° angle of refraction. To meet the group delay condition, some elements with low PCR were used in the BAMG, which resulted in the light intensity near 0° being inadequately suppressed, as shown in Figure 5d. The stars in Figure 5e represent the deflection angles predicted using Equation (2). The stars match well with the maximum simulated light intensities at the selected wavelengths, demonstrating the validity of using Equation (2) to predict the angular dispersion of the PB metasurface grating. Figure 5c shows the simulated deflection angles for the BAMG and PB metasurface grating. The deflection angle of the BAMG remained almost unchanged when the wavelength was changed from 9 μm to 11.6 μm, indicating that good achromatic performance was achieved.

## 4. Conclusions

In summary, we theoretically proposed and designed two broadband achromatic metasurfaces in the LWIR range based on an all-germanium platform, namely a BAML with NA = 0.32 and an average intensity efficiency of 31%, and a BAMG with a constant deflection angle of 30°. By combining the dynamic and PB phases, the required group delay and phase profiles were imparted to the metasurfaces simultaneously, resulting in a good achromatic performance across the entire operating bandwidth in simulations. The broadband achromatic metasurfaces can be efficiently fabricated on germanium wafers by conventional nanofabrication. We believe that the achromatic metasurfaces generated using this method will pave the way for broad applications in the LWIR, such as thermal imaging and wireless communications.

## Data Availability

The data that support the plots within this paper and other findings of this study are available from the corresponding authors on reasonable request.

## Supplementary Materials

The following are available online at https://www.mdpi.com/article/10.3390/nano11102760/s1, Figure S1: layout of PB metalens. Figure S2: SEM of the fabricated LWIR metalens. Figure S3: processing steps for germanium metalenses.
