# Supplementary material for: Broadband Achromatic Metasurfaces for Longwave Infrared Applications
Source: Nanomaterials (Basel). 2021 Oct 18;11(10):2760. doi: 10.3390/nano11102760 (PMC8538097; doi:10.3390/nano11102760)
Supplement: Supplementary file 1 [file nanomaterials-11-02760-s001.zip › nanomaterials-1394683-supplementary.pdf]

nanomaterials

MDPI

1. Layout of PB Metalens

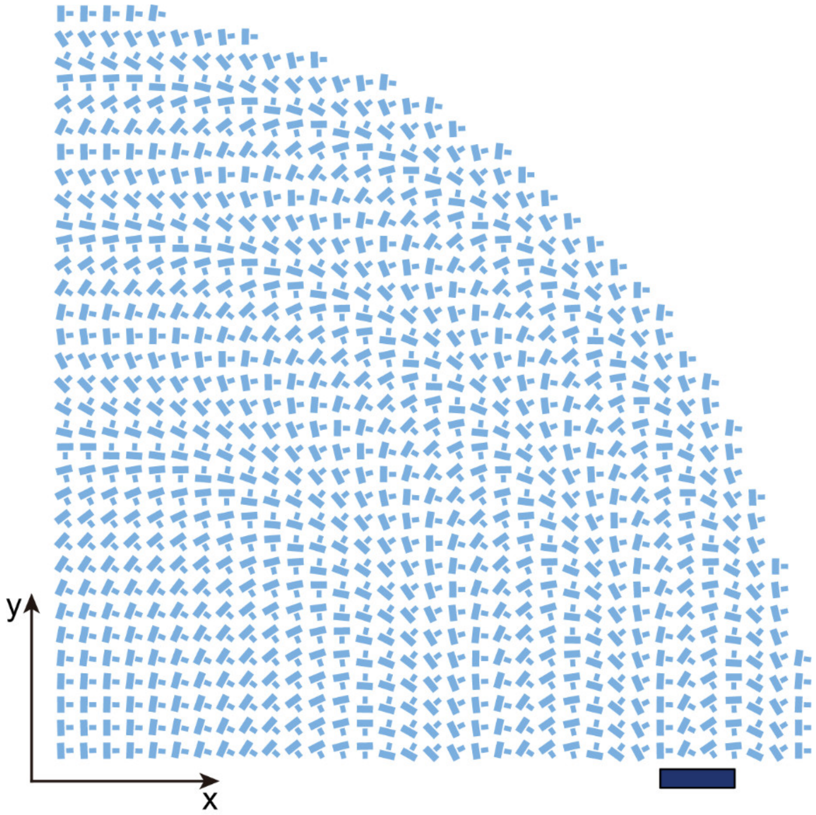

Figure S1. Layout of PB metalens.

2. Possible Methods for Processing Long-Wave Infrared Metasurfaces

Although the results in the paper are based on theoretical simulations, our proposed lens is potentially processable. As shown in Figure S2, we have tried to fabricate the LWIR metalens (composed of nanofin with circular cross section) in our lab. Because we do not have the best recipe for etching germanium, the undercutting phenomenon is very pronounced in germanium metalenses, and this phenomenon also prevents us from processing metasurfaces with large aspect ratios (the metasurface in our manuscript has a maximum aspect ratio of 10:1). The method of fabricating the silicon metalens is the same as that in [1]. To fabricate the all-germanium metalens, photoresist is first spin coated on the double-sided polished germanium wafer, then baked on a hotplate. The laser direct writing system (Heidelberg Instruments, MLA150) is used to form a metalens pattern. The pattern is then transferred to an alumina (Al2O3) mask by e-beam evaporation, then inductively coupled plasma (ICP) is used for dry etching of germanium etching with a mixture of SF6 and CHF3.

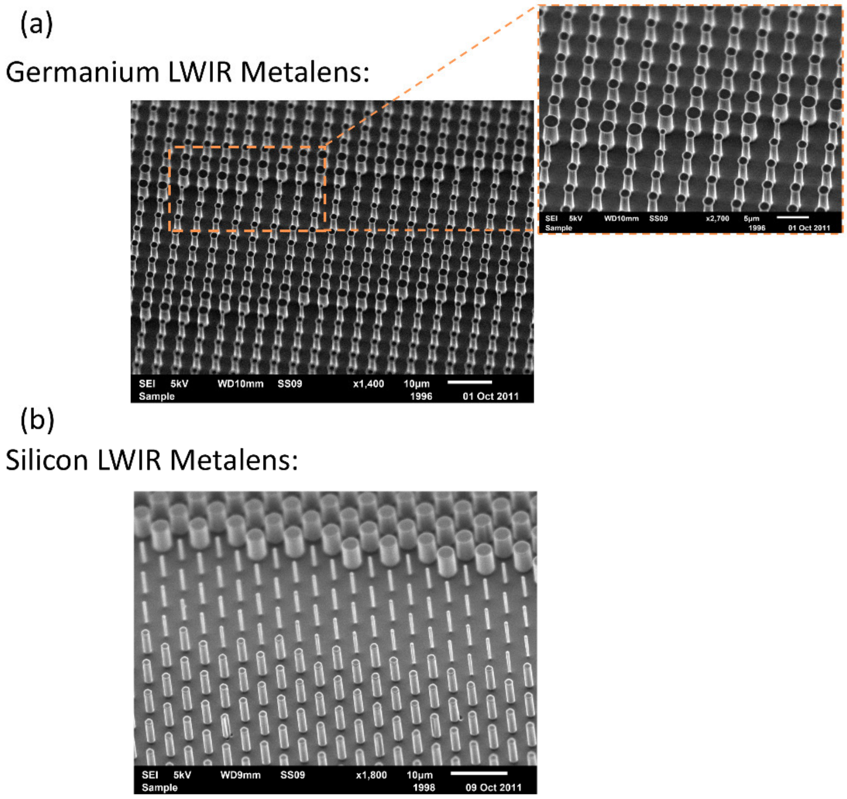

Figure S2. SEM of the fabricated LWIR Metalens. (a) Metalens based on germanium material; (b) Metalens based on silicon material.

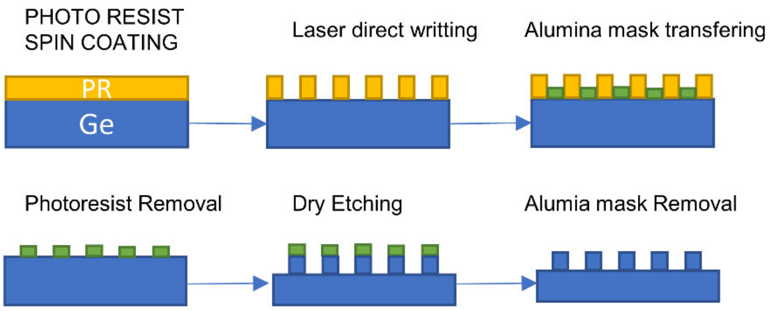

Figure S3. Processing steps for germanium metalenses.

References

1. Fan, Q.; Liu, M.; Yang, C.; Yu, L.;Yan, F. and Xu, T. A high numerical aperture, polarization-insensitive metalens for long-wavelength infrared imaging. *Appl. Phys. Lett.* **2018**, 113, 201104. https://doi.org/10.1063/1.5050562
